# Reliability and validity of the Japanese movement imagery questionnaire-revised second version

**DOI:** 10.1186/s13104-022-06220-y

**Published:** 2022-10-25

**Authors:** Hideki Nakano, Mizuki Tachibana, Nao Fujita, Shun Sawai, Shoya Fujikawa, Ryosuke Yamamoto, Shin Murata

**Affiliations:** 1 Graduate School of Health Sciences, Kyoto Tachibana University, 34 Yamada-cho, Oyake, Yamashina-ku, 607-8175 Kyoto-city, Kyoto, Japan; 2 Department of Physical Therapy, Faculty of Health Sciences, Kyoto Tachibana University, 34 Yamada-cho, Oyake, Yamashina-ku, 607-8175 Kyoto-city, Kyoto, Japan; 3 Department of Rehabilitation, Kyoto Kuno Hospital, 22-500 Honmachi, Higashiyama-ku, 605-0981 Kyoto-city, Kyoto, Japan; 4 Department of Rehabilitation, Tesseikai Neurosurgical Hospital, 28-1 Nakanohonmachi, 575-8511 Shijonawate-city, Osaka Japan

**Keywords:** Movement Imagery Questionnaire-Revised Second Version, Motor imagery, Japanese version, Reliability, Validity

## Abstract

**Objective:**

Developing a Japanese version of the Movement Imagery Questionnaire-Revised Second Version (MIQ-RS) is essential for widespread evaluation and treatment based on motor imagery in physically disabled persons and patients in rehabilitation. This study aimed to investigate the reliability and validity of the Movement Imagery Questionnaire-Revised Second Version (MIQ-RS), which assesses motor imagery ability, by translating it into Japanese.

**Results:**

This study enrolled twenty healthy participants (10 men and 10 women, mean age 21.17 ± 1.10 years). Reliability was examined for internal consistency using Cronbach’s alpha coefficient. Spearman’s rank correlation coefficient was used to examine the criterion-related validity of the MIQ-RS and the Kinesthetic and Visual Imagery Questionnaire (KVIQ-20). Results showed that Cronbach’s alpha coefficients for the MIQ-RS were 0.81 and 0.82 for visual and kinesthetic imagery, respectively. Significant positive correlations were found between each visual and kinesthetic imagery score, and each total on the MIQ-RS and KVIQ-20 scores (r = 0.73, p < 0.01; r = 0.84, p < 0.01; r = 0.80, p < 0.01, respectively). This study suggests that the Japanese version of the MIQ-RS is a reliable and valid method of assessing motor imagery ability.

**Supplementary Information:**

The online version contains supplementary material available at 10.1186/s13104-022-06220-y.

## Introduction

Motor imagery refers to the simulation of movement in the brain without actual movement [1]. It is classified into kinesthetic and visual imagery [2]. Kinesthetic imagery requires one to “feel the movement” and mentally perceive muscle contractions and stretching. However, visual imagery requires self-visualization of a movement from a first-person (internal visual imagery) or third-person (external visual imagery) perspective. The first-person perspective suggests that the participant would visualize the movement as if they had a camera on their head, representing a movement as if they were conducting the action themselves. Conversely, the third-person perspective represents movements as if the participant was a spectator and someone (either the individual or another person) performed the action. Many functional brain imaging studies have been conducted to examine their neural mechanisms [3, 4]. According to a previous study, kinesthetic imagery bilaterally activates the supplementary motor area, inferior parietal lobule, precentral gyrus, and cerebellum; the left inferior frontal gyrus, supramarginal gyrus, temporal pole, putamen, and anterior insula; and right rolandic operculum, angular gyrus, precuneus, and pallidum [3]. Moreover, visual imagery bilaterally activates the supplementary motor area, left precentral gyrus, lingual gyrus, cerebellum, and the right middle frontal and postcentral gyrus [3]. Thus, the brain regions activated by kinesthetic and visual imagery differ. Kinesthetic imagery activates motor-related regions similar to those activated by actual movement. Therefore, kinesthetic imagery has been widely applied in training and rehabilitation.

A questionnaire is a simple method of evaluating motor imagery ability. A representative questionnaire is the Movement Imagery Questionnaire-Revised, Second Edition (MIQ-RS) developed by Gregg et al. [5]. The MIQ-RS was developed based on the Movement Imagery Questionnaire (MIQ) by Hall and Pongrac in 1983 [6] and the Movement Imagery Questionnaire-Revised (MIQ-R) by Hall and Martin in 1997 [7]. The MIQ-R is a questionnaire used globally for the evaluation of motor imagery [8–10]. However, it includes items that require the subject to jump. From a physical and safety aspect, it is difficult to adapt for people with physical disabilities and patients in rehabilitation. To solve this problem, the MIQ-RS was developed; it replaces problematic items with those that reflect daily movements, making it possible to evaluate a wider spectrum of patients’ motor imagery ability.

Consequently, the MIQ-RS has been applied to the assessment of motor imagery ability in patients with stroke [11–13], traumatic brain injury [14], and amyotrophic lateral sclerosis [15], as well as in studies of athletes [16] and the brain-computer interface [17]. Furthermore, the MIQ-RS has been translated into Spanish [18] and French [19]; its reliability and validity have been verified. However, the MIQ-RS has not been translated into Japanese, and its reliability and validity have not been investigated. Developing a Japanese version of the MIQ-RS is essential for widespread evaluation and treatment based on motor imagery in physically disabled persons and patients in rehabilitation.

Assessing motor imagery ability is essential for rehabilitation and sports applications and understanding the developmental process of motor imagery and age-related changes. Body image and cognitive function, strongly related to the formation of motor imagery in infants and children, are not yet developed [20]. Conversely, the body image and cognitive functions of the elderly deteriorate with age, leading to changes in motor imagery [21]. As described above, assessing motor imagery ability is important for visualizing human developmental stages and age-related changes.

This study aimed to translate the MIQ-RS, which assesses motor imagery ability, into Japanese and to verify its reliability and validity.

## Materials and methods

### Participants

Twenty healthy participants (10 men and 10 women, mean age 21.17 ± 1.10 years, mean height 167.00 ± 8.70 cm, mean weight 60.00 ± 9.55 kg) participated in the study. The sample size was calculated using G*Power [22, 23]. G power was set as follows: test family, exact; statistical test, correlation; αerror prob, 0.05; power (1-βerror prob), 0.80. The total sample size was calculated as 19. The MIQ-RS and KVIQ have been reported to be highly correlated in a previous study [11] and were used as a reference for setting the G power. When assessed using the Edinburgh Handedness Inventory, all subjects were right-handed [24] (laterality quotient, 90.37 ± 9.61). The inclusion criteria were age ≥ 18 years, healthy status, and right-handedness. Participants with orthopedic, neurological, or psychiatric diseases that might affect the results of the study were excluded. This study was conducted after orally explaining and obtaining written consent from the participants. The study was performed considering the ethics and personal information stated in the Declaration of Helsinki. This study was approved by the local institutional ethics committee of the Kyoto Tachibana University.

### Translation procedure

The translation was performed using the forward-backward method [19]. First, two translators sequentially translated the MIQ-RS from Japanese to English. When differences in translated items in meaning or clarity arose, they were discussed and combined into one version. The forward-translated version was then back-translated from Japanese to English. The back-translated version was then reviewed, and a provisional Japanese version of the MIQ-RS was created. Finally, the provisional Japanese version of the MIQ-RS was administered to three native Japanese-speaking participants. The final Japanese version of the MIQ-RS was then created based on the feedback obtained from these participants.

### Measurement procedure

To examine the criterion-relevant validity of the MIQ-RS, participants were assessed for motor imagery ability using the MIQ-RS and Kinesthetic and Visual Imagery Questionnaire (KVIQ-20). Measurements were taken in a quiet room with the door closed.

The MIQ-RS consists of 14 items: 7 visual and 7 kinesthetic imagery items. Each item is rated on a 7-point ordinal scale, with a score of 1 indicating “Very hard to see/feel” and 7 indicating “Very easy to see/feel.” Scores for visual and kinesthetic imagery range from 7 to 49 each, and the total score ranged from 14 to 98. Supplementary material 1 Table S1 shows 14 items evaluated in the MIQ-RS [5].

The Japanese version of the KVIQ-20 [25] was used to measure the ability for imagery. It consists of 20 items: 10 visual and 10 kinesthetic imagery items. Each item is rated on a 5-point ordinal scale, with a score of 1 indicating “No image/sensation” and 5 indicating “Image as clear as seeing/As intense as executing the action.” Scores for visual and kinesthetic sensory imagery ranged from 10 to 50 each, and the total score ranged from 20 to 100.

As in previous studies [5, 25], the MIQ-RS and KVIQ-20 were measured using the following procedure: (1) The subject assumes the starting position; (2) the subject explains the movement they will perform, and performs it only once; (3) the subject returns to the starting position and imagines the movement they performed (the examiner confirms that the subject does not move during the imagery); (4) the subject evaluates the ease/difficulty of the imagined movement. The MIQ-RS uses a 7-point ordinal scale for seeing/feeling visual/kinesthetic imagery, while the KVIQ-20 uses a 5-point ordinal scale for clarity/intensity of visual/kinesthetic imagery.

### Statistical analysis

Cronbach’s alpha coefficient, a measure of internal consistency, was calculated to examine the internal consistency of the MIQ-RS. The correlation between the MIQ-RS and the KVIQ-20 was analyzed using Spearman’s rank correlation coefficient to examine criterion-related validity. SPSS 28.0 (IBM, Armonk, NY) was used for statistical analysis. The significance level was set as p < 5%.

Based on previous studies, the reference values are as follows: Cronbach’s alpha coefficients of the MIQ-RS are 0.88 for visual and 0.88 for kinesthetic imagery [18]. Spearman’s rank correlation coefficient between each visual and kinesthetic imagery scores on the MIQ-RS and KVIQ-10 (r = 0.77; r = 0.86, respectively) [11].

## Results

Twenty healthy participants (10 men and 10 women, mean age 21.17 ± 1.10 years) participated in this study. Table 1 shows the values obtained for the MIQ-RS and KVIQ-20; Cronbach’s alpha coefficients for the MIQ-RS were 0.81 and 0.82 for visual and kinesthetic imagery, respectively (Table 2). The results of the correlation between the MIQ-RS and KVIQ-20 are shown in Table 3 and Supplementary material 2 Figure S1. Significant positive correlations were found between the MIQ-RS (visual imagery) and KVIQ-20 (visual imagery), MIQ-RS (kinesthetic imagery) and KVIQ-20 (kinesthetic imagery), and MIQ-RS (total score) and the KVIQ-20 (total score) (r = 0.73, p < 0.01; r = 0.84, p < 0.01; r = 0.80, p < 0.01, respectively).

**Table 1:** Scores for the MIQ-RS and the KVIQ-20

Questionnaires	Subscale	Mean	SD
MIQ-RS	Visual (49)	40.20	4.76
	Kinesthetic (49)	32.35	6.08
	Total (98)	72.55	8.22
KVIQ-20	Visual (50)	39.45	6.64
	Kinesthetic (50)	29.35	8.20
	Total (100)	68.80	12.71

MIQ-RS, Movement Imagery Questionnaire-Revised, Second Edition. KVIQ-20, Kinesthetic and Visual Imagery Questionnaire, 20 items. MIQ-RS total scores range from 14–98 (visual and kinesthetic subscale scores each range from 7– 49). KVIQ-20 total scores range from 20–100 (visual and kinesthetic subscale scores range from 10– 50).

**Table 2:** Internal consistency of the MIQ-RS

	MIQ-RS	
	Visual	Kinesthetic
Cronbach’s α	0.81	0.82
95% CI	0.65–0.91	0.67–0.92

MIQ-RS, Movement Imagery Questionnaire-Revised, Second Edition; CI, Confidence interval.

**Table 3:** Spearman’s rank correlation coefficients between scores for the MIQ-RS and KVIQ-20

Variable	Correlation coefficient	
	**r**	***p*** **-value**
MIQ-RS Visual - KVIQ-20 Visual	0.73	< 0.01
MIQ-RS Kinesthetic - KVIQ-20 Kinesthetic	0.84	< 0.01
MIQ-RS Total - KVIQ-20 Total	0.80	< 0.01

MIQ-RS, Movement Imagery Questionnaire-Revised, Second Edition. KVIQ-20, Kinesthetic and Visual Imagery Questionnaire, 20 items.

## Discussion

The present study was conducted to verify the reliability and validity of the MIQ-RS, which assesses motor imagery, by translating it into Japanese. The results showed that the MIQ-RS had high internal consistency. It showed a significant positive correlation with the KVIQ-20. These results indicate that the Japanese version of the MIQ-RS is a reliable and valid questionnaire assessing motor imagery ability.

The mean MIQ-RS values for the visual imagery, kinesthetic imagery, and total scores in this study were 40.20, 32.35, and 72.55, respectively. The visual imagery, kinesthetic imagery, and total scores for the MIQ-RS Spanish version in healthy individuals were 39.61, 36.08, and 75.69, respectively [18]. Furthermore, the results of this study were similar to those of the English and French versions of MIQ-RS in healthy participants [5, 11, 19]. Conversely, the mean KVIQ-20 values in the present study were 39.45, 29.35, and 69.80 for visual imagery, kinesthetic imagery, and total scores, respectively. A previous study reported that each KVIQ-20 score in healthy participants for visual imagery, kinesthetic imagery, and total scores was 37.00, 37.21, and 74.21, respectively [25], and the results obtained in this study were similar to the previous study. Therefore, the results of MIQ-RS and KVIQ-20 obtained in this study adequately reflected the participants’ motor imagery ability, similar to previous reports.

This study examined internal consistency using Cronbach’s alpha coefficient to measure reliability. The results showed that Cronbach’s alpha coefficients of MIQ-RS were 0.81 for visual and 0.82 for kinesthetic imagery. Cronbach’s alpha coefficients for MIQ-RS in other language versions are reported as follows: English version, 0.98 for visual and 0.97 for kinesthetic imagery [11]; Spanish version, 0.88 for visual and 0.88 for kinesthetic imagery [18]; French version, 0.90 for the whole [19]. Moreover, internal consistency was assessed by estimating the standardized Cronbach’s alpha coefficient, which is generally considered acceptable with a coefficient > 0.7, good at a minimum of 0.8, and excellent when superior to 0.9 [11]. Our results were similar to those reported in previous studies [11, 18, 19], and the present results indicate that the Japanese version of MIQ-RS has high reliability as an index for assessing motor imagery ability.

This study also examined criterion-related validity as a measure of validity. Significant positive correlations were found between the MIQ-RS and KVIQ-20 visual imagery, kinesthetic imagery, and total scores. Previous studies have validated the criterion-related validity of the English version of MIQ-RS using MIQ-R and KVIQ-10, and have reported significant positive correlations [5, 11]. The MIQ-RS criterion-related validity obtained in this study was similar to that of previous studies. Therefore, the MIQ-RS Japanese version was shown to have high validity as an index for evaluating motor imagery ability.

The MIQ-RS scores in this study were higher for visual imagery than that for kinesthetic imagery. Higher scores for visual imagery than kinesthetic imagery was also observed in the English, Spanish, and French versions of the MIQ-RS [5, 11, 18, 19], which may be due to attentional focus superiority. Sakurada et al. reported that individuals with visual imagery predominance are more likely to focus their attention on the external body, while individuals with kinesthetic imagery predominance are more likely to focus on the internal body [26, 27]. These studies have also revealed that the group, which was more likely to pay attention to the internal body (kinesthetic imagery dominant), had higher improvement in motor performance [26, 27]. Application of kinesthetic imagery in the rehabilitation and sports fields [28, 29] suggests that attentional focus dominance is related to characteristics of visual and kinesthetic imagery.

## Conclusion

This study investigated the reliability and validity of the MIQ-RS in Japan, which assesses motor imagery ability, by translating it into Japanese. Results showed that the MIQ-RS had high internal consistency, and a significant positive correlation was found between the MIQ-RS and KVIQ-20. This study indicates that the Japanese version of the MIQ-RS is a reliable and valid method of assessing motor imagery ability. This is expected to contribute to the evaluation and treatment of motor imagery in physically disabled persons and patients in rehabilitation.

## Limitations

There are some limitations in this study. First is that it only included healthy participants. Therefore, the usefulness of the Japanese version of the MIQ-RS could not be examined for persons with physical disabilities. However, studies of the MIQ-RS in stroke [10–12], traumatic brain injury [13], and amyotrophic lateral sclerosis [14] have been reported in the past. In future, the usefulness of the Japanese version of the MIQ-RS should be validated for people with physical disabilities and patients undergoing rehabilitation. Additionally, it is necessary to consider the use of MIQ-RS and KVIQ, depending on the degree of functional impairment and the case pathophysiology. Second, the sample size is small. Future studies should be conducted with a larger sample size to examine reliability and validity in more detail. Third, the off-diagonal correlation of visual and muscular sensory imagery is unknown in the present study. Future studies should also analyze how the subscales of motor imagery are related.

## Electronic supplementary material

Below is the link to the electronic supplementary material.

Supplementary Material 1 Table S1

Supplementary Material 2 Figure S1

## Data Availability

The data that support the findings of this study are available on request from the corresponding author. The data are not publicly available because they contain information that can compromise the privacy of research participants.

## Abbreviations

MIQ-RS: Movement Imagery Questionnaire-Revised, Second Edition
KVIQ-20: Kinesthetic and Visual Imagery Questionnaire, 20 items
