# Supplementary material for: Reliability and validity of the Japanese movement imagery questionnaire-revised second version
Source: BMC Res Notes. 2022 Oct 25;15:334. doi: 10.1186/s13104-022-06220-y (PMC9594881; doi:10.1186/s13104-022-06220-y)
Supplement: Supplementary file 1 — Supplementary Material 1 Table S1 [file 13104_2022_6220_MOESM1_ESM.pdf]

Table S1. 14 items evaluated in the MIQ-RS [5]

Item	Subscale	Action
1	KI	Raise your one knee as high as possible so that you are standing on one leg with your other leg flexed (bent) at the knee. Now lower your leg so that you are again standing on two feet.
2	VI	Raise your hand above your head until your arm is fully extended, keeping your fingers in a fist. Next, lower your hand back to your lap while maintaining a fist.
3	KI	Move your arm forward until it is directly in front of your body (still parallel to the ground). Keep your arm extended during the movement and move slowly. Now move your arm back to the starting position, straight out to your side.
4	VI	Slowly bend forward at the waist and try and touch your toes with your fingertips. Now return to the starting position, standing erect with your arms extended above your head.
5	VI	Extend your arm fully as if you are pushing open the door, keeping your fingers pointing upwards. Now let the swinging door close by returning your hand and arm to the starting position.
6	KI	Reach forward, grasp the glass, and lift it slightly off the table. Now place it back on the table and return your hand to your lap.
7	KI	Reach forward, grasp the door handle, and pull open the door. Now gently shut the door, let go of the door handle and return your arm to your side.
8	VI	Raise your one knee as high as possible so that you are standing on one leg with your other leg flexed (bent) at the knee. Now lower your leg so that you are again standing on two feet.
9	KI	Raise your hand above your head until your arm is fully extended, keeping your fingers in a fist. Next, lower your hand back to your lap while maintaining a fist.
10	VI	Move your arm forward until it is directly in front of your body (still parallel to the ground). Keep your arm extended during the movement and move slowly. Now move your arm back to the starting position, straight out to your side.
11	KI	Slowly bend forward at the waist and try and touch your toes with your fingertips. Now return to the starting position, standing erect with your arms extended above your head.
12	KI	Put your hand in front of you at about shoulder height as if you are about to push open a swinging door. Your fingers should be pointing upwards.
13	VI	Reach forward, grasp the glass, and lift it slightly off the table. Now place it back on the table and return your hand to your lap.
14	VI	Reach forward, grasp the door handle, and pull open the door. Now gently shut the door, let go of the door handle and return your arm to your side.

VI, Visual Imagery; KI, Kinesthetic Imagery.
