# Supplementary material for: Reliability and validity of the Japanese movement imagery questionnaire-revised second version
Source: BMC Res Notes. 2022 Oct 25;15:334. doi: 10.1186/s13104-022-06220-y (PMC9594881; doi:10.1186/s13104-022-06220-y)
Supplement: Supplementary file 2 — Supplementary Material 2 Figure S1 [file 13104_2022_6220_MOESM2_ESM.pdf]

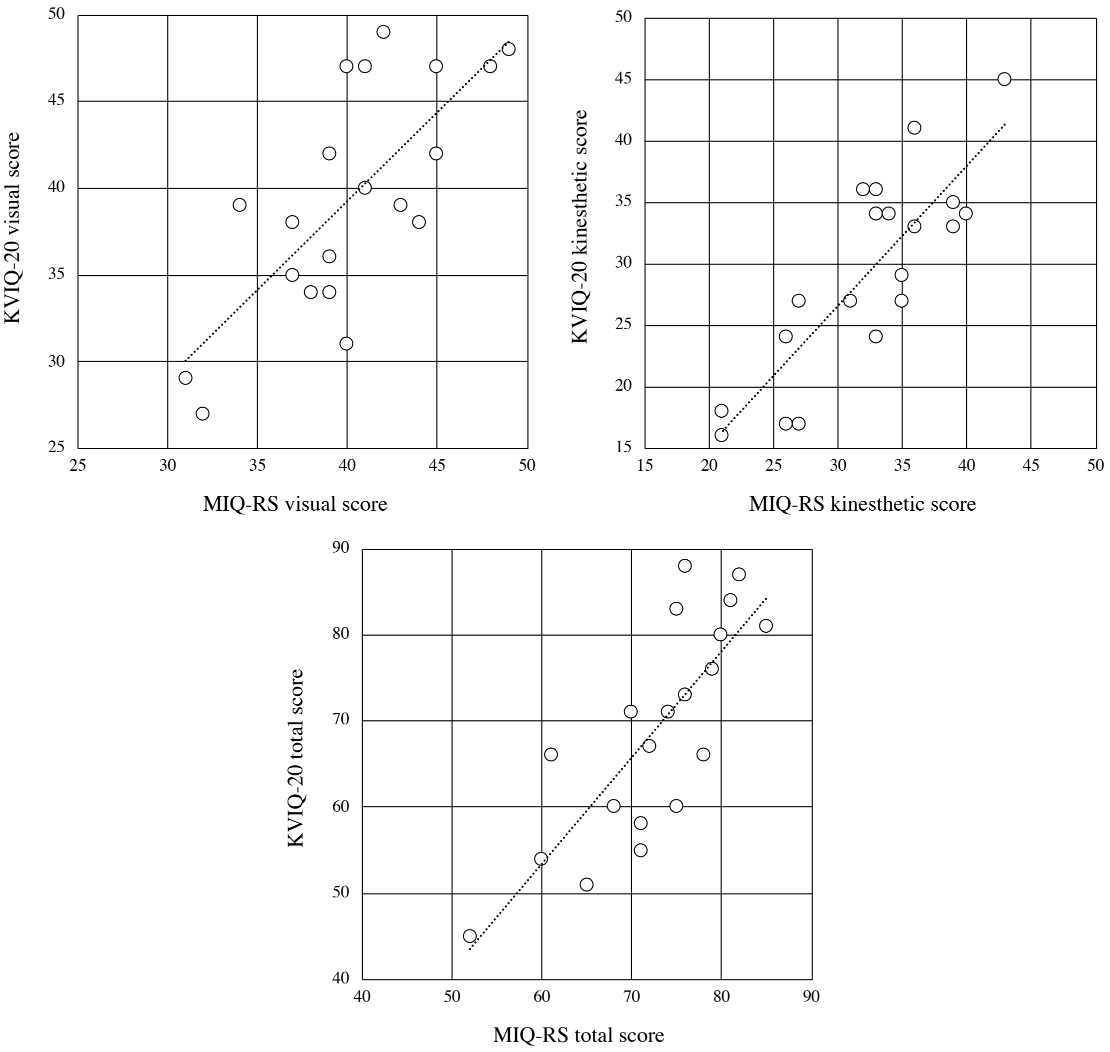

Figure S1. Spearman's rank correlation coefficients between scores for the MIQ-RS and KVIQ-20

Significant positive correlations were found between the MIQ-RS (visual imagery) and KVIQ-20 (visual imagery), MIQ-RS (kinesthetic imagery) and KVIQ-20 (kinesthetic imagery), and MIQ-RS (total score) and the KVIQ-20 (total score).
